# A coarse-grained resource allocation model of carbon and nitrogen metabolism in unicellular microbes

**DOI:** 10.1098/rsif.2023.0206

**Published:** 2023-09-27

**Authors:** Istvan T. Kleijn, Samuel Marguerat, Vahid Shahrezaei

**Affiliations:** ^1^ Department of Mathematics, Faculty of Natural Sciences, Imperial College London, London, UK; ^2^ Institute of Clinical Sciences, Faculty of Medicine, Imperial College London, London, UK; ^3^ MRC London Institute of Medical Sciences, London, UK; ^4^ Division of Breast Cancer Research, The Institute of Cancer Research, London, UK; ^5^ Ralph Lauren Centre for Breast Cancer Research, The Royal Marsden NHS Foundation Trust, London, UK

**Keywords:** microbial growth, mathematical modelling, proteome allocation, metabolism, cellular growth laws, gene expression

## Abstract

Coarse-grained resource allocation models (C-GRAMs) are simple mathematical models of cell physiology, where large components of the macromolecular composition are abstracted into single entities. The dynamics and steady-state behaviour of such models provides insights on optimal allocation of cellular resources and have explained experimentally observed cellular growth laws, but current models do not account for the uptake of compound sources of carbon and nitrogen. Here, we formulate a C-GRAM with nitrogen and carbon pathways converging on biomass production, with parametrizations accounting for respirofermentative and purely respiratory growth. The model describes the effects of the uptake of sugars, ammonium and/or compound nutrients such as amino acids on the translational resource allocation towards proteome sectors that maximized the growth rate. It robustly recovers cellular growth laws including the Monod law and the ribosomal growth law. Furthermore, we show how the growth-maximizing balance between carbon uptake, recycling, and excretion depends on the nutrient environment. Lastly, we find a robust linear correlation between the ribosome fraction and the abundance of amino acid equivalents in the optimal cell, which supports the view that simple regulation of translational gene expression can enable cells to achieve an approximately optimal growth state.

## 1. Introduction

Unicellular organisms are remarkably efficient self-replicators as they are under selective pressure to grow fast or risk being outcompeted by rival colonies or species [1,2]. On the other hand, microbial cells are faced with internal constraints limiting their growth, because each metabolite, macromolecule, or unit of membrane area can only be used for one reaction at any given time. Fast-growing cells must therefore possess an ability to allocate these limited resources in varied environments [3–6]. The interplay between gene expression and growth can be studied reproducibly in states of balanced growth, where cells are maintained in the same environment for many generations [7].

Observed patterns of gene expression and the resulting rate of growth depend heavily on the growth environment, in particular on the presence of external stresses and, importantly, the nutrient make-up. A well-established feature of gene expression in multiple model organisms is the presence of linear correlations between broad classes of macromolecules and the cellular growth rate [8–12]. The most pervasive positive correlation is between the abundance of translational resources (chiefly ribosomes) and the growth rate [13–17]. Classes of proteins that are negatively correlated with the growth rate are associated with stress, or induced by the specific cause of growth inhibition—stressors or reductions in the nutrient quality or quantity [15,17–19].

These observed correlations between the total abundance of proteome sectors and the cellular growth rate have been explored in several coarse-grained phenomenological and mechanistic models [20–28]. These coarse-grained resource allocation models (C-GRAMs) make explicit the intuition that the conversion of nutrient into biomass can be done in more or less efficient ways. Abundant proteins are costly to produce and therefore have a large effect on the growth rate, which is a measure of fitness [6].

Extended summaries of these models are provided in electronic supplementary material, text S1. All were used to study trade-offs between resource allocation towards metabolic versus ribosomal gene expression and they all account for the experimental relation between ribosomal gene expression and the growth rate. Another common feature of these earlier models is the hyperbolic dependence of the growth rate on the concentration of external nutrients, observed first experimentally in [29]. C-GRAMs have been used to model the burden of synthetic circuits in the host cell [24,26]. Another powerful application of the coarse-grained approach came from [27], which explained how differences in cell size across different growth modulations could be explained by the underlying proteome composition.

The coarse-graining approach of C-GRAMs entails that large sectors of the proteome are abstracted into a single protein, whose kinetics are explicitly described. The coarse-graining approach boils down assumptions about metabolism, growth, gene expression and cellular physiology into only a handful of parameters. Fitting nonlinear models is a challenging problem in general and, in the case of whole-genome models, further complicated by the large number of parameters to be estimated. By contrast, a coarse-grained approach is much more computationally tractable than explicitly accounting for the complexity of metabolism. This is particularly true for minimal approaches such as proposed in [20], which constructed a C-GRAM that incorporated nitrogen metabolism and described proteome allocation under optimal growth, and [27], which additionally included metabolites into the system size.

Because of their tractability, C-GRAMs are also well suited to exploring hypotheses about the interplay between metabolism, growth and gene expression [30], and finding insights on the general principles behind the physiology of unicellular organisms [6]. They can furthermore be designed in an organism-agnostic manner and thereby provide an opportunity to compare microbes based on those model parametrizations that best explain global observations about growth in each organism. In summary, the coarse-grained approach allows one to directly interpret and explore model parameters, with minimal need for explicit or large-scale parameter inference.

Earlier C-GRAMs chiefly considered carbon modulations representative for the effect of the nutrient quality in general. In such models, metabolism was typically considered as a linear pathway from nutrient to protein production. This included the models proposed in [24] and in [27]. Contrasting with this earlier work, a strategy commonly employed in *Schizosaccharomyces pombe* to modulate the growth rate uses ammonium chloride and a variety of amino acids as nitrogen sources [17,31]. Using this strategy, we have recently reported differential expression for many enzymes involved in carbon metabolism across nitrogen sources, even though abundant glucose was provided in all conditions [17]. Different amino acids have also been used as sole nitrogen sources in order to modulate growth while studying the proteome of *Saccharomyces cerevisiae* [32] and of three bacterial strains found in the *Arabidopsis* rhizosphere [33].

We aimed to better understand the effect of nitrogen-source modulation on resource allocation in a coarse-grained modelling context. We took a minimal approach, opting to construct a model with fewer parameters and therefore omitting transcription, the distinction between energy and amino acid metabolism. We extended the metabolic model proposed in [27] to include the uptake and metabolism of carbon-containing nitrogen sources. We studied how a growth-rate-maximizing allocation towards proteome sectors varied between steady states that were imposed by choices of parameters representing different nutrient conditions.

## 2. Results

In this study, we aim to investigate optimal resource allocation behaviour under growth in defined media with varying nitrogen sources. This, for example, will be relevant for growth media where different amino acids act as the sole source of nitrogen. Amino acids generally consist of an amino group and a ketoacid backbone, and we therefore focused on modelling nitrogen and carbon metabolism. We constructed a C-GRAM with pathways representing nitrogen and carbon uptake, protein biosynthesis, and the excretion and recycling of carbon from carbon-containing nitrogen sources. The model was formulated dynamically using ordinary differential equations (ODEs), their steady state representing balanced growth. The steady-state growth rate was calculated from the solution to the ODEs as an emergent property.

Pathways in the model were represented by enzymes with simple kinetics, and the parameters in this representation were chosen to reflect nutrient conditions. For example, carbon limitation was modelled by reducing the catalytic rate of the carbon uptake enzyme. Furthermore, nitrogen sources were distinguished by the catalytic rates of the enzymes metabolizing them as well as their stoichiometries, related to their elemental carbon-to-nitrogen ratio. For each nutrient condition (parametrization), we determined the resource allocation that maximized the growth rate. The details regarding the model implementations, namely the full formulation of the ODEs, considerations with regards to parameter choices, and the approach used to optimize resource allocation are described in the Methods.

As described in the remainder of this manuscript, we explored the effect of different growth-rate modulations on the optimal allocation. We first considered the behaviour of two submodels that lacked the recycling and excretion of the ketoacid backbone. This enabled us to explore trade-offs in resource allocation in media with simple sources of carbon (such as glucose) and nitrogen (such as ammonium chloride). In the first submodel, no distinction was yet made between respirofermentative and purely respiratory metabolism; in the second submodel, two parallel pathways representing the two were introduced. Finally, the full model was used to describe growth on amino-acid nitrogen sources. As expected, various nutrient limitations induced complex trade-offs between allocations to different enzyme fractions.

### 2.1. Modulation of the carbon uptake rate with one metabolic pathway

**Figure 1.**
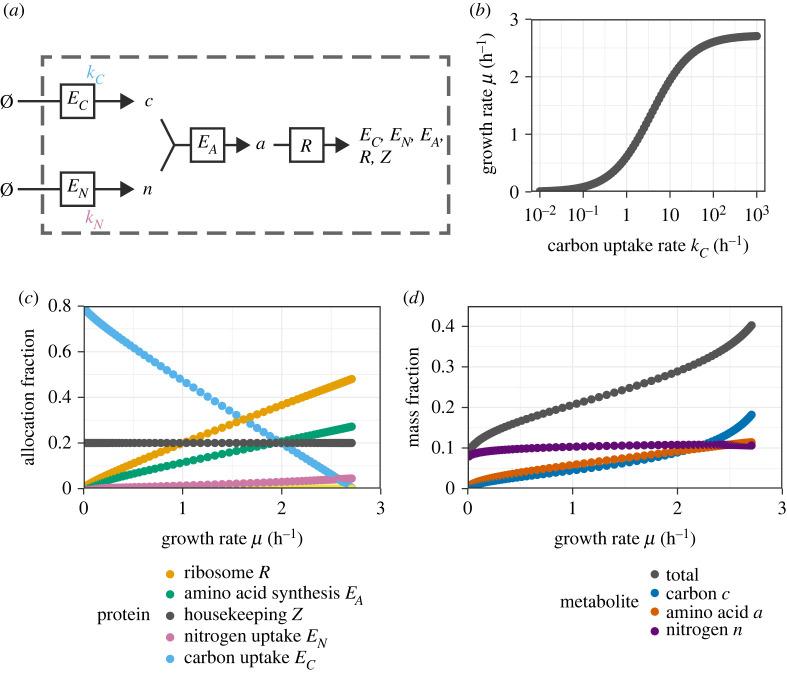
Analysis of the core metabolic model under modulation of the carbon uptake rate *k*_*C*_. The uptake rate parameter *k*_*C*_ of the transporter was modulated to yield different growth rates. The allocation fractions were chosen to maximize the growth rate for each chosen *k*_*C*_. (*a*) Illustration of the model. (*b*) Growth rate *μ* as a function of the carbon uptake rate *k*_*C*_. (*c*) Optimal protein allocation fractions as a function of the growth rate *μ*. (*d*) Steady-state mass fractions of metabolites as a function of the growth rate *μ*.

The first submodel we explored was one with parallel uptake of carbon and nitrogen, which were combined by a single amino-acid-producing enzyme. An illustration of the model is provided as figure 1*a* and the interpretation of all model constituents (proteins and metabolites) is provided in table 1. This model was explored by varying the carbon uptake rate parameter *k*_*C*_. All model parameters are listed in table 2.

**Table 1.** Variables (metabolites and proteins) implemented in the full model.

type	description	concentration	allocation fraction
metabolite	ketoacid	*k*	n.a.
	carbon	*c*	
	amino acid	*a*	
	free nitrogen	*n*	
protein	ketoacid recycler	$e_{K_{\mathrm{re}}}$	$f_{K_{\mathrm{re}}}$
	ketoacid excreter	$e_{K_{\mathrm{ex}}}$	$f_{K_{\mathrm{ex}}}$
	carbon uptake	*e*_*C*_	*f*_*C*_
	respirofermentative enzyme	$e_{A_{f}}$	$f_{A_{f}}$
	purely respiratory enzyme	$e_{A_{r}}$	$f_{A_{r}}$
	nitrogen-compound uptake	*e*_*N*_	*f*_*N*_
	ribosome	*r*	*f*_*R*_
	housekeeping (non-metabolic)	*z*	*f*_*Z*_

**Table 2.** Parameters (Michaelis constants, enzyme efficiencies and stoichiometries) implemented in the full model.

type	description	symbol	default value
Michaelis constant	ketoacid	*k*_sat_	0.0167
	carbon	*c*_sat_	0.0167
	amino acid	*a*_sat_	0.0167
	free nitrogen	*n*_sat_	0.0167
enzyme efficiency	ketoacid recycler	$k_{K_{\mathrm{re}}}$	10.0 h^−1^
	ketoacid excreter	$k_{K_{\mathrm{ex}}}$	20.0 h^−1^
	carbon uptake	*k*_*C*_	10.0 h^−1^
	respirofermentative enzyme	$k_{A_{f}}$	15.0 h^−1^
	purely respiratory enzyme	$k_{A_{r}}$	7.5 h^−1^
	nitrogen-compound uptake	*k*_*N*_	20.0 h^−1^
	ribosome	*k*_*R*_	6.46 h^−1^
mass stoichiometry	carbon consumed per amino acid produced in respirofermentative metabolism	$\alpha_{C_{f}}$	48/55
	carbon consumed per amino acid produced in purely respiratory metabolism	$\alpha_{C_{r}}$	24/31
	ketoacid produced per nitrogen compound consumed	*γ*_*K*_	0

As expected, the dependency of the growth rate on the modulation parameter approximated a Monod curve (figure 1*b*), and the optimal protein allocation fractions were approximately linearly correlated with the growth rate (figure 1*c*). The total relative metabolite abundance in steady state amounted to approximately 10%–30% of biomass (figure 1*d*). In experimental cultures, metabolites comprise only a small fraction of the biomass (BNID 111490; [34,35]), so in this respect the growth-optimized model was a poor approximation for conditions where this occurred. This may reflect the current parametrization of the model to be inaccurate.

Importantly, under modulation of the carbon uptake enzyme, the allocation fractions of the nitrogen assimilation enzyme *E*_*N*_ and the amino-acid biosynthesis enzyme *E*_*A*_ were positively correlated with the growth rate (figure 1*c*). This behaviour was also implemented in [20], but it is notably different from the model described in [24], which assumed that the transporter and metabolic enzyme were both regulated identically.

Proteins associated with translation are estimated to constitute approximately 45% of the total protein mass in the fastest-growing *Escherichia coli* cultures [13,15,19], which reach maximal growth rates of around 2.0 h^−1^. A minimal partitioning of the proteome based on growth rate correlations suggested that around half of the proteome mass does not change with the growth rate [13,21]. The earlier model in [24] reflected this by implementing protein expression regulation with a metabolic sector, a translational sector and a constitutive sector. Its proteome comprised two sequential enzymes (corresponding to our *E*_*C*_ and *E*_*A*_), ribosomes (our *R*), and housekeeping proteins (our *Z*). In their parametrization, the latter took up around 70% of the total protein mass, and were negatively correlated with the growth rate.

By contrast, in the parametrization used to generate figure 1, the allocation towards housekeeping proteins was only 20% of the proteome. However, this parametrization was chosen such that the maximal ribosomal allocation agreed reasonably well with *E. coli* data from [13]. The discrepancy between the two models in their estimated housekeeping allocations is explained by the considerable cost of the non-modulated enzymes. At maximal growth, the amino-acid synthesis and nitrogen-uptake enzymes amount to around 25% and 5% of the proteome, respectively.

### 2.2. Modulation of carbon and nitrogen uptake rates with two parallel metabolic pathways

Next, we considered that a key determinant of unicellular growth is the metabolic state, with a principal difference whether fermentation occurs or whether biosynthesis is maintained in a purely respiratory fashion. Fast-growing cells express both fermentative and respiratory pathways, with the former the primary generator of free energy (in the form of ATP). When growing on poorer or less abundant carbon sources, cells rely on respiration for energy generation, but respiratory enzymes and the citric acid cycle are involved in amino acid biosynthesis even when free energy is primarily generated by fermentation [36]. Comparing the two pathways at equivalent energy generation, the fully respiratory pathway poses a greater burden on gene expression. On the other hand, the respirofermentative pathway requires more carbon uptake from the environment, because it is converted into overflow metabolites such as acetate or ethanol. We aimed to construct a minimal model allowing for these two different metabolic states, and wondered how the optimal state would be affected by nitrogen and carbon limitation.

Deciding against introducing further metabolites and enzymes to the model, we instead implemented two parallel amino-acid synthesis pathways. The parametrization of these parallel pathways reflected the above considerations about carbon uptake and gene expression burden. First, we implemented the purely respiratory enzyme *E*_*Ar*_ with a lower enzyme efficiency than the respirofermentative *E*_*Af*_. Second, we adjusted the carbon stoichiometry, such that *E*_*Af*_ required additional carbon per nitrogen atom consumed (eight atoms instead of four; for details, see Methods §4.3), representing overflow metabolism without implementing it as an additional pathway. The allocation fractions to both enzymes were included in the growth rate optimization. Our parametrization implied that *E*_*Ar*_ required less carbon substrate, but a higher expression to sustain the same synthesis rate, than *E*_*Af*_.

**Figure 2.**
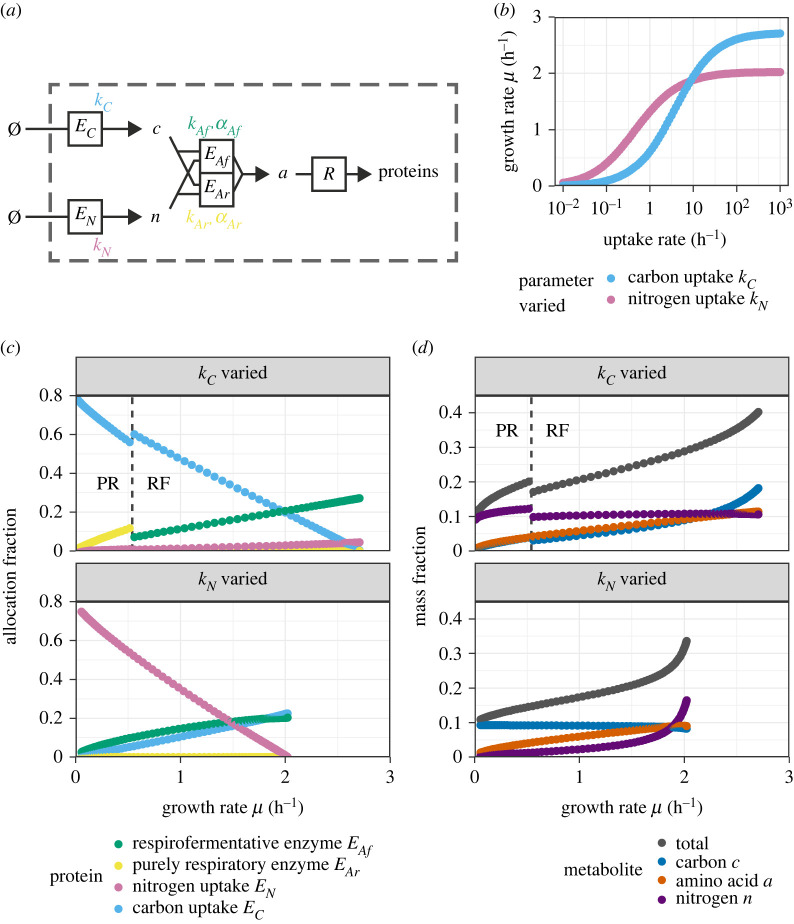
Analysis of the model with two parallel metabolic enzymes (respirofermentative enzyme *E*_*Af*_ and purely respiratory enzyme *E*_*Ar*_) under the separate modulation of the carbon and nitrogen transporter uptake rates *k*_*C*_ and *k*_*N*_, respectively. Allocation fractions were chosen to maximize the growth rate for each chosen *k*_*C*_ (*c* and *d*, top panels) and *k*_*N*_ (bottom panels). The uptake rates were varied separately between 1.0 × 10^−2^ and 1.0 × 10^3^ h^−1^. (*a*) Illustration of the model. (*b*) Growth rate *μ* as a function of the respective uptake rate. (*c*) Optimal protein allocation fractions as a function of growth rate *μ* for the two parameter explorations. Ribosomes *R* and housekeeping proteins *Z* were omitted from the figure to improve clarity. (*d*) Steady-state mass fractions of metabolites as a function of growth rate *μ*. The transition between purely respiratory (PR) and respirofermentative (RF) growth is indicated by a dashed line.

We modulated the carbon and nitrogen transporter rate parameters in the model containing two parallel metabolic pathways with different parameterizations (figure 2*a*). As before, the growth rate depended on the modulation parameters approximately following a Monod curve (figure 2*b*). Because the two pathways are parallel, the usage of one of the two was preferred over the other in each parametrization, such that one of *E*_*Ar*_ and *E*_*Af*_ was expressed while the other was not. As shown in figure 2*c*, the purely respiratory enzyme was induced by low values of the carbon uptake rate *k*_*C*_. We note a sharp and discontinuous transition from respiration to fermentation at a specific level of carbon uptake rate (figure 2*c*). This agrees with results of a model similar in scope to ours, where the optimal choice between a metabolically efficient or catabolically efficient pathway was shown to depend on the growth rate [20]. In contrast to this behaviour under modulations of the carbon uptake, the respirofermentative enzyme was present in all conditions where the nitrogen uptake efficiency *k*_*N*_ was varied from its default.

Both the internal carbon abundance *c* and the nitrogen abundance *n* varied discontinuously with the growth rate near the transition point (*μ* ≈ 0.5^−1^ h). This indicates how purely respiratory metabolism required a higher abundance of both of its substrates (*c* and *n*) than respirofermentative metabolism to sustain the same turnover. By contrast, the abundance of the product, the amino acid equivalent *a*, varied almost continuously with the growth rate even at the transition point. The relation between *a* and the ribosomal allocation will be explored in detail in a further section of this paper.

Under perturbations of the nitrogen transporter uptake rate *k*_*N*_ (figure 2*c*, bottom panel), the allocation towards the metabolic enzyme varied nonlinearly with the growth rate. In addition to this, the amount of nitrogen metabolite *n* built up much more strongly with increasing growth rate via *k*_*N*_ than the equivalent carbon build-up under perturbations of *k*_*C*_ (figure 2*d*). Note that modulating each transporter’s efficiency (*k*_*N*_ and *k*_*C*_) repressed the abundance of its own substrate (*n* and *c*, respectively). On the other hand, the modulation of one transporter rate barely affected homeostasis of the metabolite in the other pathway (*c* and *n*, respectively).

### 2.3. Recycling and excretion of ketoacids disturbed carbon metabolism

In the above, we restricted ourselves to the metabolism of freely usable nitrogen, which typically comes in the form of ammonium ions (${\mathrm{NH}}_{4}^{\;\;+}$). However, the cellular growth rate can also be perturbed considerably by using different nitrogen sources, particularly amino acids, as the sole nitrogen source instead of ${\mathrm{NH}}_{4}^{\;\;+}$ [17,31]. These pathways typically deaminate or transaminate the amino acid, either by a single enzyme or as the net result of a longer pathway [37]. Nitrogen is assimilated as free ${\mathrm{NH}}_{4}^{+}$ (after deamination) or glutamate (after transamination). The leftover carbohydrate, usually a ketoacid, may be recycled into biomass or excreted.

**Figure 3.**
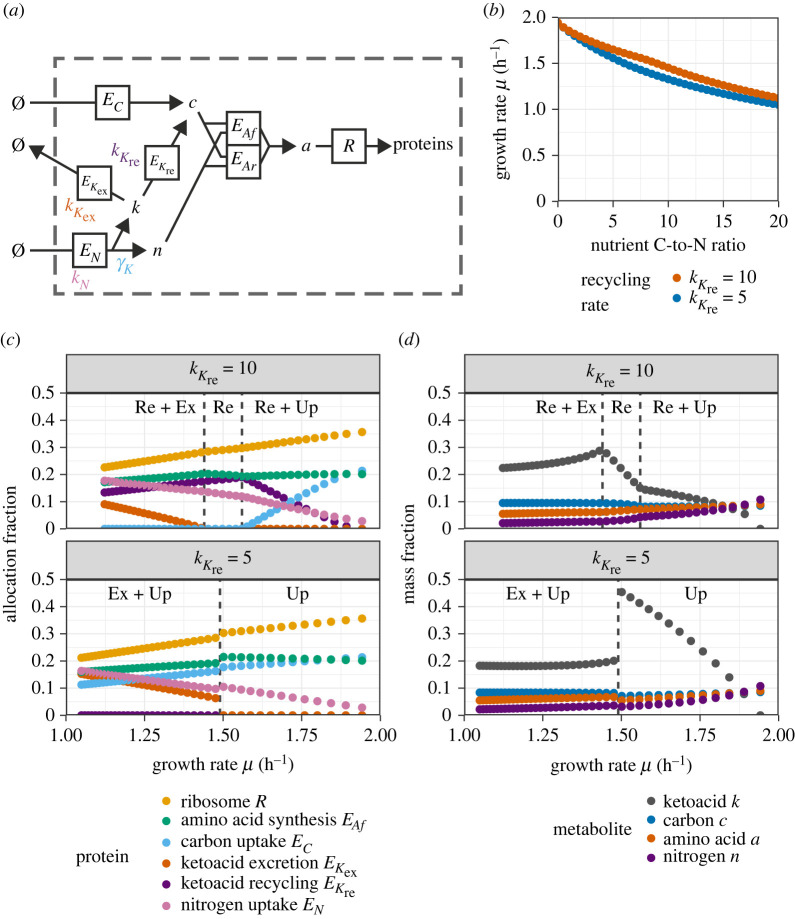
Analysis of the full model, including recycling and excretion of ketoacid under modulation of the carbon-to-nitrogen ratio in the nitrogen source for two choices of the ketoacid recycling rate $k_{K_{\mathrm{re}}}$. The carbon-to-nitrogen ratio was varied between 0 and 20 in steps of 0.5 and the ketoacid stoichiometry *γ*_*K*_ was calculated from this as described in the text. Allocation fractions were chosen to maximize the growth rate for each *γ*_*K*_ and $k_{K_{\mathrm{re}}}$. (*a*) Illustration of the model. (*b*) Growth rate *μ* as a function of the nitrogen source’s carbon-to-nitrogen (C-to-N) ratio for the respective ketoacid recycling rates. (*c*) Optimal protein allocation fractions as a function of growth rate *μ* for the two recycling rates. Purely respiratory enzymes *E*_*Ar*_ and housekeeping proteins Z were omitted from the figure to improve clarity; *E*_*Ar*_ was not expressed for any of the parametrizations shown. (*d*) Steady-state mass fractions of metabolites as a function of growth rate *μ*. Transitions between metabolic states are indicated by dashed lines (Re, ketoacid recycling; Ex, ketoacid excretion; Up, carbon uptake).

To account for this process in the model, we implemented the nitrogen uptake pathway as an enzyme that produced both free nitrogen and a carbon-containing ketoacid metabolite. To represent different complex nitrogen sources such as amino acids, we perturbed the parameter *γ*_*K*_, which represents the relative mass of recycled ketoacid with respect to the total mass taken up by the nitrogen uptake enzyme. Additionally, we introduced two enzymes, *E*_*K*re_ and *E*_*K*ex_ that, respectively, recycled the ketoacid into usable carbon precursor or excreted it from the cell. The model is illustrated in figure 3*a*.

The behaviour of the model that included ketoacids depended qualitatively and quantitatively on the values of key parameters, particulary on the efficiencies (catalytic rates) of the enzymes involved and on the ketoacid stoichiometry *γ*_*K*_. Recycling enzymes were only expressed if their efficiency (catalytic rate) $k_{K_{\mathrm{re}}}$ was large. We first explored the model behaviour for two choices of this parameter, modulating *γ*_*K*_ but leaving fixed all other metabolic parameters. The results are shown in figure 3*b*–*d*.

Based on the types of proteins that were expressed, there were three qualitatively different growth regimes for $k_{K_{\mathrm{re}}}=10$ and two for $k_{K_{\mathrm{re}}}=5$. One important trade-off here is between the cellular cost of expressing ketoacid processing enzymes and the cost of carrying the excess metabolite for cell growth. When recycling enzymes were inefficient and costly ($k_{K_{\mathrm{re}}}=5$, bottom panels), but the carbon-to-nitrogen ratio was still below a certain threshold, the ketoacid metabolites built up considerably (up to almost half the total biomass). In this regime, this still led to faster growth than expressing either recycling or excretion enzymes would have. However, with the amino acid nitrogen source containing relatively more carbon, the growth-optimized cell-excreted ketoacids and the internal concentration were approximately stable across the range of growth rates investigated. When recycling enzymes were efficient ($k_{K_{\mathrm{re}}}=10$, top panels), recycling replaced the uptake of carbon through the canonical carbon pathway, i.e. no carbon uptake enzyme *E*_*C*_ was expressed below a critical growth rate. For both choices of $k_{K_{\mathrm{re}}}$, in the fastest-growing regime, no excretion took place, i.e. the excretion enzyme *E*_*K*ex_ was not expressed; but in the slowest-growing regime, ketoacid excretion was required to optimize growth. Curiously, we found a recycling-only regime with neither canonical carbon uptake nor ketoacid excretion. In this latter regime, all carbon in the biomass has its origin in the amino acid from the nutrient, and all the carbon from the nutrient made its way into the cell.

We note that across all simulations, no respiratory enzyme *E*_*Ar*_ was expressed. Furthermore, the optimal allocation to ribosomal proteins deviated discontinuously from the ribosomal growth law for both choices of $k_{K_{\mathrm{re}}}$ when the ketoacid stoichiometry *γ*_*K*_ was varied.

### 2.4. Different nutrient environments (parametrizations) induced complex trade-offs between carbon uptake, ketoacid recycling and excretion

We further explored the five different growth regimes across a more extensive parameter sweep. The ketoacid stoichiometry *γ*_*K*_ is related to the ratio of carbon and nitrogen atoms in a nutrient molecule

2.1 $$\gamma_{K}=\frac{n_{C}\times 12}{n_{C}\times 12+n_{N}\times 14}=\frac{12(n_{C}/n_{N})}{12(n_{C}/n_{N})+14},$$

where *n*_*C*_ and *n*_*N*_ are the numbers of carbon and nitrogen atoms in a nutrient molecule, and the factors 12 and 14 account for the approximate molar masses of these two elements. For example, glycine molecules contain two carbon atoms for each nitrogen atom, which would be represented by *γ*_*K*_ = 2 × 12/(2 × 12 + 14) = 12/19 ≈ 0.63, whereas each molecule of isoleucine contains nine carbon atoms, for *γ*_*K*_ = 9 × 12/(9 × 12 + 14) = 54/61 ≈ 0.89.

**Figure 4.**
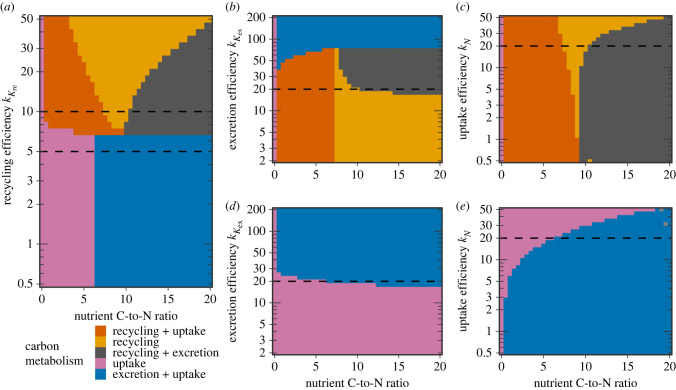
Phase diagrams of ketoacid recycling, excretion and carbon uptake under perturbations of enzyme efficiencies and nutrient carbon-to-nitrogen ratio. For each figure, the optimal allocation was determined for 41^2^ combinations of the ketoacid stoichiometry *γ*_*K*_ and one of the enzyme efficiencies $k_{K_{\mathrm{re}}}$, $k_{K_{\mathrm{ex}}}$ and *k*_*N*_. As before, the carbon-to-nitrogen ratio was varied between 0 and 20 in steps of 0.5 and the ketoacid stoichiometry *γ*_*K*_ was subsequently calculated. Furthermore, the enzyme efficiencies were chosen to be equidistant after log-transformation. In the figure, colours indicate whether ketoacid recycling, ketoacid excretion and carbon uptake enzymes were expressed in the optimal allocation, and dashed lines indicate parameter values that were fixed in the other panels and in figure 3. (*a*) Ketoacid recycling rate $k_{K_{\mathrm{re}}}$ varied, ketoacid excretion rate $k_{K_{\mathrm{ex}}}=20.0\;h^{-1}$ and nutrient uptake rate *k*_*N*_ = 20.0 h^−1^ held fixed. (*b*) $k_{K_{\mathrm{ex}}}$ varied, $k_{K_{\mathrm{re}}}=10.0\;h^{-1}$ and *k*_*N*_ = 20.0 h^−1^ fixed. (*c*) *k*_*N*_ varied, $k_{K_{\mathrm{re}}}=10.0\;h^{-1}$ and $k_{K_{\mathrm{ex}}}=20.0\;h^{-1}$ fixed. (*d*) and (*e*) as *b* and *c*, but with $k_{K_{\mathrm{re}}}=5.0\;h^{-1}$.

Alongside the ketoacid stoichiometry *γ*_*K*_, we varied the maximal turnover rates (efficiencies) *k*_*N*_, $k_{K_{\mathrm{ex}}}$ and $k_{K_{\mathrm{re}}}$ of the enzymes involved in ketoacid metabolism, as plotted in figure 4. This showed that the five regimes highlighted in the previous section were universal. The optimally allocated cell expressed only one or two out of the ketoacid recycling, ketoacid excretion, and carbon uptake enzymes depending on the parametrization (figure 3). Neither recycling nor excretion was expressed in nitrogen sources not also containing carbon, and additionally this was optimal even for carbon-containing nitrogen sources when the ketoacid recycling and excretion enzymes were inefficient relative to the nitrogen uptake enzyme (figure 4*a*,*d*,*e*). The value of the ketoacid recycling rate $k_{K_{\mathrm{re}}}$ below which ketoacid recycling was suboptimal depended weakly on the ketoacid stoichiometry (figure 4*a*). When recycling enzymes were expressed, the trade-off between the excretion and ketoacid uptake was heavily influenced by the nitrogen source’s carbon content and all three enzyme rates (figure 4*a*–*c*). Low-carbon nutrient sources required additional carbon uptake whereas high-carbon nutrient sources generally required ketoacid excretion, with pure recycling being favoured in regimes with intermediate carbon content or inefficient excretion.

### 2.5. Approximately optimal allocation towards ribosomes could result from amino-acid regulation

Until now, we have explored the model using defined modulations of one or more rate parameters and the ketoacid stoichiometry. Across all of these, we found that the ribosomal growth law (ribosomal proteome allocation fraction *f*_*R*_ ∝ *μ*) was robustly satisfied. Moreover, the amino acid abundance *a* appeared to be similarly correlated with the growth rate. We wondered if this was an artefact of our parametrization approach or a deeper property of the model and used a random sampling parametrization strategy to further study this behaviour. We sampled 100 triplets of the rate parameters $\left(k_{C},k_{N},k_{A_{f}}\right)$ from independent uniform distributions with support [0, 20], set $k_{A_{r}}=0.5k_{A_{f}}$, and matched these samples to four choices of the ketoacid stoichiometry *γ*_*K*_, representing carbon-to-nitrogen ratios of 0, 3, 6 and 12, and two choices of the ketoacid recycling efficiency $k_{K_{\mathrm{re}}}$.

**Figure 5.**
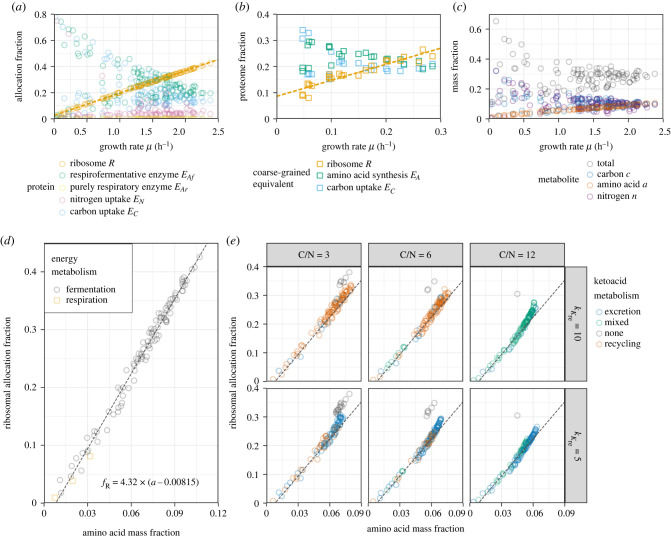
Analysis of the full model with randomly chosen rate parameters, exploring the relation between amino acid precursor abundance and ribosomal allocation. The enzyme efficiencies $k_{A_{f}}$, *k*_*C*_ and *k*_*N*_ were randomly drawn from separate uniform distributions ranging between 0 and 20.0 m^−1^, and the respiratory enzyme efficiency $k_{A_{r}}$ was set to $0.5k_{A_{f}}$. The ketoacid recycling efficiency $k_{K_{\mathrm{re}}}$ and carbon-to-nitrogen ratio were initially fixed to 10.0 h^−1^ and 0, respectively (for *a*, *c* and *d*), and then varied jointly (for *e*). The allocation fractions were chosen to maximize the growth rate for each set of parameters. (*a*) Optimal protein allocation fractions as a function of the growth rate *μ*. Housekeeping proteins and ketoacid-processing enzymes were omitted to improve clarity. The best-fit of a linear regression model to the ribosomal allocation fraction is drawn as a dashed line to guide the eye. (*b*) Experimentally observed proteome mass fractions from a *Schizosaccharomyces pombe* study, mapped to their coarse-grained equivalents. (*c*) Steady-state biomass fractions of amino acid *a*, carbon *c*, and free nitrogen *n* plotted against the growth rate *μ*. (*d*) Scatter plot of the amino acid concentrations *a* and ribosomal allocation fractions *f*_*R*_, indicating the type of energy metabolism that optimized growth (fermentation: only *E*_*Af*_ expressed; respiration: only *E*_*Ar*_ expressed). The dashed line represents an ordinary least-squares linear model fit to the data. (*e*) As *d* for three non-zero choices of the carbon-to-nitrogen ratio (C/N) and two choices of the ketoacid recycling efficiency *k*_*K*re_. Points are coloured according to whether none, one, or both of the ketoacid excretion enzyme *E*_*K*ex_ and ketoacid recycling enzyme *E*_*K*re_ was expressed. The dashed line indicates the fit from *d* (no further fitting was performed here).

In the ketoacid-free model (*γ*_*K*_ = 0) with random rate parameters, the ribosomal growth law was nearly exactly satisfied (figure 5*a*), even though expression of the two transporters and the metabolic enzyme was highly variable between parameter choices. In a recent experimental study, we also observed this contrast between the linear ribosomal growth law and variable expression of metabolic classes [17]. In figure 5*b*, we show the expression of protein classes mapped to their three C-GRAM equivalent: ribosomes (translation and ribosome biogenesis from [17]), carbon uptake (glycolysis), and amino acid synthesis (precursors and energy generation, and amino acid metabolism).

Furthermore, the concentration of the amino acid *a* was closely related to growth rate as well (figure 5*c*). It follows from the growth rate correlations for both *f*_*R*_ and *a* that the two were correlated themselves as well (figure 5*d*). A linear fit *f*_*R*_ ∝ (*a* − *a*_0_) closely approximated the observed relation.

We further explored the relation between *f*_*R*_ and *a* in the presence of complex nitrogen sources such as amino acids, when additionally the recycling and/or excretion of ketoacid is accounted for (figure 5*d*). For large carbon-to-nitrogen ratios, the relation between *f*_*R*_ and *a* may be better described by a nonlinear relation, although the fit for *f*_*R*_ ∝ (*a* − *a*_0_) obtained for nitrogen sources without carbon was still a close approximation. Only when the optimal solution involved ketoacids building up in the cell without recycling or excretion did the linear fit break down entirely. Since our model does not account for the toxicity of the intermediate ketoacids beyond their passive drag on growth, this situation is not likely to occur in real cells.

All together with the previous sections, these results suggest that a single linear regulation of ribosomes by amino acids can achieve near optimal allocation of ribosomes. For the regimes explored here, a simple proportionality *f*_*R*_ = *δa* such as proposed in [27] would work reasonably well, though our introduction of an offset *a*_0_ (i.e. *f*_*R*_ = *δ*(*a* − *a*_0_)) improved the fit. Interestingly, if *f*_*R*_ ∝ *a* is chosen and the ribosomes are implemented with Michaelis–Menten kinetics, the ribosomal growth law follows analytically. A derivation of this statement is presented in electronic supplementary material, text S4; it holds whether or not there is an offset *a*_0_ in the *f*_*R*_–*a* relationship. We note that we did not observe a simple relationship between the allocation of other non-ribosomal proteins and the metabolite concentrations.

## 3. Discussion

A holistic understanding of growth, gene expression and resource allocation can be codified in and achieved by C-GRAMs. Here, we constructed a minimal C-GRAM of microbial metabolism that accounted for (i) the metabolism of both carbon and nitrogen, (ii) the different proteomic and kinetic efficiencies of respirofermentative and respiratory energy metabolism and (iii) the stoichiometry of complex nitrogen sources that contain a carbon backbone, such as amino acids. We optimized the allocation towards different protein classes so as to maximize the growth rate.

Different parameter sweeps of the model allowed us to explore different nutrient environments. The optimal allocation and resulting growth rate co-varied according to strikingly regular behaviour. In particular, the Monod law and ribosomal growth law were very robust. Furthermore, we described growth on carbon-containing nitrogen sources by introducing the possibility of recycling and/or excreting residual carbon, which induced complex trade-offs in metabolism. Notably, both the optimal allocation towards ribosomes and their substrate (internal amino acids) varied approximately linearly with the growth rate, leading to an also approximately linear relationship between the two.

### 3.1. Protein reserves and simple feedback of free amino acids setting ribosome allocation

Our assumption that expression of all proteins was optimized for growth in any given condition considerably simplified the model parametrization. Precise optimality conditions have been formulated for the *E. coli* carbon uptake system and gene expression indeed maximized the growth rate for several carbon sources [38]. Furthermore, a recent theoretical advance pointed out a general method of adapting gene expression control towards the optimum [39]. However, recent evidence has challenged the view that all allocation is growth-optimal. It is thought that significant fractions of the *E. coli* [40–42] and budding yeast [16,43] proteome are not necessary for sustaining the growth rate. In particular, central carbon metabolism has been suggested to have a large reserve capacity, suggesting that many enzymes are not used solely to maximize metabolic fluxes [32,44,45]. Pools of proteins held in reserve may be beneficial instead by their ability to support adaptation to environmental changes.

An intermediate step between a fully growth-optimized and a dynamically regulated allocation model may be the proportional regulation *f*_*R*_ ∝ *a*. We showed this to be a good approximation to the growth-rate-maximizing allocation; additionally, it was robust to many variations of the parameters describing the nutrient quality. The simplicity of this relation is remarkable: in principle, optimal allocation could depend on all internal metabolite concentrations and be highly nonlinear, instead of this linear dependence on only a single metabolite. Explicit regulation of bacterial ribosome synthesis mediated by a single metabolite, guanosine tetraphosphate (ppGpp), was thoroughly explored in a coarse-grained model by [46]. Recently, ppGpp was shown to regulate the growth rate and ribosome content in *E. coli* by sensing the instantaneous translation elongation rate [47].

Notably, neither our growth-optimized model nor one implementing ribosomal allocation proportional to internal amino acids explicitly account for a reserve pool of ribosomes not actively involved in translation. Such a pool has been held responsible for the observed offset *ϕ*_*R*0_ in the ribosomal growth law *ϕ*_*R*_ = *ϕ*_*R*0_ + *σ*^−1^*μ* [16,42,48]. Although our model did not implement any inactive ribosomes, we still observed an offset greater than zero. We explain this effect by the fact that the ribosomes in our model are not fully saturated with substrate, even when growth is optimized. Specifically, linearly correlated ribosome allocation and amino-acid abundance (either through optimization or explicit regulation), combined with nonlinear Michaelis–Menten ribosome kinetics, resulted in a offset

3.1 $$\phi_{R0}=\delta a_{\mathrm{sat}},$$

when *f*_*R*_ = *δ*(*a* − *a*_0_) and *f*_*R*_ ∝ *a*/(*a* + *a*_sat_). Naturally, ribosome saturation and inactivation are not mutually exclusive, and a quantitative understanding of their relative importance will have profound implications on our understanding of the interplay between ribosome synthesis, excess translational capacity and cell growth [49].

### 3.2. The fate of the carbon backbone for complex nitrogen sources such as amino acids

We next discuss the fate of the carbon backbone from complex nitrogen sources, as modelled first in the model presented here. In our model, modulations of the ketoacid stoichiometry *γ*_*K*_ gave rise to a wide range of growth rates in a monotonically decreasing manner. However, a nitrogen source’s quality is not solely determined by its carbon-to-nitrogen ratio. For example, glycine and tryptophan media gave rise to very similar growth rates in *S. pombe* ([17], but carbon is present in a 2 : 1 ratio in glycine and in a 5.5 : 1 ratio in tryptophan (see electronic supplementary material, text, table S2). This strongly suggests that each growth medium is not only associated with the ketoacid stoichiometry *γ*_*K*_, but that at least one out of the enzymatic rates *k*_*N*_ (nitrogen uptake), $k_{K_{\mathrm{ex}}}$ (ketoacid excretion) and $k_{K_{\mathrm{re}}}$ (ketoacid recycling) must also be modulated by the choice of nitrogen source.

Unlike translation and central carbon metabolism, the topology of amino acid uptake pathways is rather poorly conserved between organisms. In practice, then, whether recycling or excretion is preferred for a given amino acid nitrogen source depends on which reactions are available to the organism, and how efficient they are. If ketoacid recycling effectively feeds into other synthesis pathways, this would correspond to a large $k_{K_{\mathrm{re}}}$ in the model; on the other hand, the effectiveness (or absence) of suitable excretion pathways would influence the value of $k_{K_{\mathrm{ex}}}$.

While our model provides a framework for understanding the optimality of recycling and excretion of carbon, we refrained from a full parametrization of growth on specific nitrogen sources. We note that despite this limitation, our model agrees with metabolic gene expression being medium-specific rather than correlated with growth rate, as has indeed been recently reported in *S. pombe* growing on different amino acids (figure 5*a*,*b*).

In terms of possible biotechnological applications, we note that carbon uptake from complex nitrogen sources may be advantageous from a yield perspective. However, its effect on the growth rate is generally deleterious as reflected by the model (see e.g. figure 3*b*). As our model shows, choosing a complex nitrogen source will affect carbon metabolism, though it probably will not repress fermentation. To study the interplay between metabolism and the expression of synthetic constructs, our C-GRAM could be extended by implementing an additional coarse-grained protein in the manner of [24].

Understanding the balance between carbon uptake, recycling and excretion is also important for applications where the product consists mostly of excretions, such as brewing. The metabolism of indigestible ketoacids has been well studied in *S. cerevisiae*, whose excretions of such ‘fusel oil’ can spoil the product [37]. While the carbon-to-nitrogen ratio is specific to the nutrient, our results shown in figure 4 suggest that the balance between excretion and recycling is not only affected by the respective efficiencies of these two processes itself, but also the uptake efficiency. In other words, if a complex nitrogen source contains relatively much carbon, it may still be effectively recycled if it is otherwise efficiently assimilated.

To get a better idea of the exact molecules and pathways involved, these general considerations should be supplemented with whole-genome models of metabolism. It is challenging to condense those into C-GRAMs due to the complexity of metabolism; the presence of many parallel pathways, moonlighting enzymes and metabolic cycles are just three examples of this complexity. However, some progress may be made in adapting our generic C-GRAM to specific organisms. Recently, high-quality metabolic maps that are aware of limited resource allocation in multiple cellular compartments have been developed for *S. cerevisiae* and *S. pombe* [50,51]. Such maps may enable comparisons between the coarse-grained proteome sectors and proteome data in the future.

### 3.3. Explicit overflow metabolism and energy generation

We accounted for the distinction between purely respiratory and respirofermentative growth by adjusting parameters of the enzyme representing the pathway, similar to one approach in [20]. Specifically, we adjusted *α*_*C*_, which represents the stoichiometry of carbon required for biomass production relative to nitrogen, and *k*_*E*_, the efficiency (maximal specific flux) of the pathway. This induced differing behaviours under nitrogen and carbon limitation: carbon limitation induced a switch to fully respiratory growth upon decreasing growth rates, but nitrogen limitation did not. However, an important feature of fermentation, namely the excretion of overflow metabolites, was not explicitly modelled. Furthermore, we did not include the generation of cellular energy in the form of ATP. These two omissions are related, as ATP is generated in different amounts by fermentative and respiratory pathways. A natural extension of the model would therefore be the addition of an explicit fermentative pathway, and parametrizing metabolic pathways by relative amounts of ATP generation and consumption.

In this light, we note that our model predicts sharp and discontinuous changes in gene expression and metabolite abundances where the switch between optimal metabolic strategies occurs (figure 2*c*,*d*). Because the substrates and products of the model were identical, our fully respiratory and respirofermentative enzymes could function as drop-in replacements of each other. Therefore, expression of either one or the other was optimal in our system. Such behaviour has been observed earlier in a similar C-GRAM framework [20] with linear carbon metabolism. There, adding explicit ATP metabolism-induced mixed expression of metabolically and catabolically efficient pathways.

Related to this, Basan *et al.* [52] studied fermentative fluxes under growth rate modulations in *E. coli*. They experimentally observed no fermentation below a critical growth rate, whereas fluxes increased rapidly with growth rate above the critical point. All results were quantitatively explained by a model of efficient proteome allocation balancing the need for biomass synthesis and energy generation. The finite resolution of the data does not enable distinguishing true discontinuity from a continuous transition over a narrow range of growth rates. What is clear is that fermentation does not completely replace respiration but rather supplements it at fast growth. This discussion highlights how C-GRAM proteins such as our fermentorespiratory enzyme must be interpreted cautiously as they represent multiple metabolic responsibilities.

### 3.4. Non-protein biomass

A second caveat to our choice modelling biosynthesis in one simple pathway is the following. While the parameter *α*_*C*_ represented the biomass carbon-to-nitrogen ratio in the model, it cannot be directly equated to observed dry mass compositions in real cells (some examples are collated in electronic supplementary material, text, table S3). This is partially due to the inclusion of extra carbon that real cells excrete during fermentation (see previous paragraph and electronic supplementary material, text S4.3), but also because the biomass of real cells consists of additional macromolecules besides proteins. For example, nucleotides are important cellular constituents with a higher nitrogen content than proteins, and an efficient cell must synthesize them in proportion to ribosomal proteins, as most RNA is ribosomal. Upstream, nucleotide synthesis depends on the pentose phosphate pathway (PPP), which shares multiple intermediate metabolites with glycolysis and amino acid metabolism. The trade-off between glycolytic and PPP flux has been implied to influence the relationships between growth rate, yield and oxidative stress [45,53,54]. A coarse-grained model that includes both protein and nucleotide synthesis must account for the coordination between the two carbon metabolic pathways [55], considerably increasing its complexity versus the model presented in this study.

Another large contribution to the biomass of microbes comes from their cell surface, which mostly consists of carbon. The interplay between cell surface biosynthesis, size homeostasis and growth has been explored in coarse-grained models of bacteria [56–58]. The relative importance of the cell surface changes with the size and shape of the cell, both of which depend on growth conditions and fluctuate during the cell cycle as recently reported in fission yeast [59]. Therefore, the dry mass density varies as cell mass and cell volume evolve differently. While balanced growth may be defined in terms of repeated cell cycles and the dynamic equations may be studied outside of steady state, the fluctuating dry mass density invalidates a focus on relative abundances. Accounting for the contribution of the cell surface to biomass in our model would therefore require reformulating the model into absolute abundances and including the effect of the cell cycle, which was outside the scope of this study. However, we expect that the improved understanding of concepts such as the optimization of resource allocation, the kinetic modelling of coarse-grained pathways, and the parametrization of stoichiometries, as presented in this work, should be useful in the development of surface-aware C-GRAMs.

### 3.5. Conclusion

In summary, we have presented a modelling framework that describes uptake and metabolism of carbon and nitrogen in unicellular microbes in a coarse-grained manner. While the parametrization of the model presented here was chosen to facilitate comparisons with earlier models of *E. coli*, the structure of the model was deliberately kept general. The framework may therefore be applied to study the optimality of gene expression and growth across the tree of life. We hope that extensions of our model will be constructed to describe overflow metabolism, ATP, nucleotide metabolism, a cell surface and/or cell density fluctuations.

## 4. Methods

### 4.1. Dynamic equations describing the metabolic model

The model from [27] served as our starting point. We disregarded non-metabolic proteins and the inhibition of ribosomes, which were both present in the original model, because here we aimed to describe the steady-state behaviour of unperturbed wild-type cultures. We then introduced additional metabolic pathways representing (i) the uptake of carbon and nitrogen, (ii) fermentative and respiratory energy generation, represented by different parametrizations of a similar enzyme, and (iii) the recycling and excretion of carbon from complex nitrogen sources containing both nitrogen and carbon. Simplifications of the model were obtained by setting some model parameters to zero.

The reactions included in the full model are pictured in figure 3*a*; the interpretations of the variables and parameters in the model are given in tables 1 and 2. These reactions were modelled by the following formalism of ODEs, which is explored in more detail in electronic supplementary material, text S2. The time evolution of the concentration vector x was decomposed and given by

4.1 $$\dot{x}=\left(\begin{array}{c} \dot{a}\\ \dot{m}\\ \dot{p} \end{array}\right)=Sj(x)-\mu x\left(\begin{array}{c} S^{A}\cdot \hat{j}(x)-j_{R}(x)-\mu a\\ \hat{S}\hat{j}(x)-\mu m\\ j_{R}(x)f-\mu p \end{array}\right).$$

The concentrations described by this equation are those representing the amino acids *a*, the other metabolites m=(k,c,n), and the proteins $p=(e_{K_{\mathrm{re}}},e_{K_{\mathrm{ex}}},e_{C},e_{A_{\;f}},e_{A_{r}},e_{N},r,z)$. The ribosomal allocation is given by the vector $f=(f_{K_{\mathrm{re}}},f_{K_{\mathrm{ex}}},f_{C},f_{A_{\;f}},f_{A_{r}},f_{N},f_{R},f_{Z})$ (table 1). Fluxes catalysed by the proteins are given by the vector

4.2 $$j=\left(\begin{array}{c} \;j_{K_{\mathrm{re}}}\\ j_{K_{\mathrm{ex}}}\\ j_{C}\\ j_{A_{f}}\\ j_{A_{r}}\\ j_{N}\\ j_{R} \end{array}\right)=\left(\begin{array}{c} k_{K_{\mathrm{re}}}e_{K_{\mathrm{re}}}\frac{k}{k+k_{\mathrm{sat}}}\\ k_{K_{\mathrm{ex}}}e_{K_{\mathrm{ex}}}\frac{k}{k+k_{\mathrm{sat}}}\\ k_{C}e_{C}\\ k_{A_{\;f}}e_{A_{f}}\frac{cn}{\left(c+c_{\mathrm{sat}})(n+n_{\mathrm{sat}}\right)}\\ k_{A_{r}}e_{A_{r}}\frac{cn}{\left(c+c_{\mathrm{sat}})(n+n_{\mathrm{sat}}\right)}\\ k_{N}e_{N}\\ k_{R}r\frac{a}{a+a_{\mathrm{sat}}} \end{array}\right).$$

The enzymes were chosen to follow Michaelis–Menten kinetics (for single-substrate enzymes) and products thereof, such that the rate laws were linear in the enzyme concentration, linear in the substrate concentrations at low concentrations, and saturated at high substrate concentrations. Details on the enzyme kinetics are provided in electronic supplementary material, text S3. Note that the housekeeping proteins (with concentration *z*) do not catalyse any enzymatic reaction and therefore did not feature in the fluxes (table 2). Finally, the stoichiometry matrix was given by

4.3 $$S=\left(\begin{array}{c} {\left(S^{A}\right)}^{T}\\ \hat{S} \end{array}\right)=\left(\begin{array}{cccccc} 0 & 0 & 0 & +1 & +1 & 0\\ -1 & -1 & 0 & 0 & 0 & \gamma_{K}\\ 1 & 0 & 1 & -\alpha_{C_{f}} & -\alpha_{C_{r}} & 0\\ 0 & 0 & 0 & -\alpha_{N_{f}} & -\alpha_{N_{r}} & \gamma_{N} \end{array}\right).$$

Mass balance was maintained in all internal reactions, meaning columns in the stoichiometry matrix representing these totalled zero. However, the carbon and nitrogen transporters imported nutrients from the environment (so columns totalled +1), and the ketoacids could be excreted to the environment by the respective enzyme (so this column totalled −1). These considerations imposed the following constraints:

4.4 $$\alpha_{C_{f}}+\alpha_{N_{f}}=1,$$

4.5 $$\alpha_{C_{r}}+\alpha_{N_{r}}=1$$

and

4.6 $$\gamma_{K}+\gamma_{N}=1.$$

Using the above definitions, the ODEs describing the system can be written as

4.7 $$\dot{a}=j_{A_{\;f}}+j_{A_{r}}-j_{R}-\mu a,$$

4.8 $$\dot{k}=\gamma_{K}j_{N}-j_{K_{\mathrm{re}}}-j_{K_{\mathrm{ex}}}-\mu k,$$

4.9 $$\dot{c}=j_{C}+j_{K}-\alpha_{C_{f}}j_{A_{f}}-\alpha_{C_{r}}j_{A_{r}}-\mu c,$$

4.10 $$\dot{n}=\gamma_{N}j_{N}-\alpha_{N_{f}}j_{A_{f}}-\alpha_{N_{r}}j_{A_{r}}-\mu n,$$

4.11 $${\dot{e}}_{K}=f_{K}j_{R}-\mu e_{K},$$

4.12 $${\dot{e}}_{C}=f_{C}j_{R}-\mu e_{C},$$

4.13 $${\dot{e}}_{A_{f}}=f_{A_{f}}j_{R}-\mu e_{A_{f}},$$

4.14 $${\dot{e}}_{A_{r}}=f_{A_{r}}j_{R}-\mu e_{A_{r}},$$

4.15 $${\dot{e}}_{N}=f_{N}j_{R}-\mu e_{N},$$

4.16 $$\dot{r}=f_{R}j_{R}-\mu r$$

4.17 $$\;\mathrm{and}\;\dot{q}=f_{Q}j_{R}-\mu q.$$

The growth rate *μ* is found by taking the sum of these equations. Furthermore, using the allocation constraint $\sum_{i}f_{i}=1$ and the concentration constraint $\sum_{i}x_{i}=1$, such that $\sum_{i}{\dot{x}}_{i}=0$, gives

4.18 $$\mu =j_{C}+j_{N}-j_{K_{\mathrm{ex}}}=k_{C}e_{C}+k_{N}e_{N}-k_{K_{\mathrm{ex}}}e_{K_{\mathrm{ex}}}\frac{k}{k+k_{\mathrm{sat}}}.$$

This is equal to the net import of nutrient from the environment (uptake minus excretion).

### 4.2. Balanced growth

As mentioned in the main text, we aimed to describe gene expression allocation under balanced growth in defined environments. The state of balanced growth was represented in the model by the steady state of equation (4.1). To compute the steady state numerically, we evolved these equations until a steady state was reached.

Given a model parametrization (see §4.3), an initial condition was partially guessed from the allocation fractions. Because the protein content in steady state is proportional to the proteome allocation, the initial relative abundance of each protein was chosen as a fixed multiple of its allocation fraction parameter. Furthermore, each initial relative metabolite abundance was established manually as 0.05. All together, the initial concentration vector $x_{0}$ was set to

4.19 $$x_{0}=\left(\begin{array}{c} a_{0}\\ m_{0}\\ p_{0} \end{array}\right)=\left(\begin{array}{c} a_{0}\\ \left(k_{0},c_{0},n_{0}\right)\\ p_{0} \end{array}\right)=\left(\begin{array}{c} 0.05\\ \left(0.05,0.05,0.05\right)\\ 0.8f \end{array}\right),$$

with ***f*** the given allocation vector.

The simulations were implemented in the Julia programming language, using the DifferentialEquations.jl ecosystem [60]. For solving the concentration ODEs towards steady state, we used the Rodas5 solver.

### 4.3. Parametrization

Unicellular organisms grow in one of two principal states: respirofermentative growth and entirely respiratory growth. From the point of view of coarse-grained modelling, there are two primary differences between the two. On the one hand, the fermentation pathway consists of few different enzymes. Although the individual enzymes are highly abundant, they are highly efficient. The aggregate effect on the total expression burden is that the fermentation enzymes comprise a markedly smaller proteome fraction than the respiratory pathway would when providing equal biomass and energy production. On the other hand, fermentation requires more nutrients from the environment: carbon is consumed rapidly and converted into ethanol or acetate. We encoded the efficiency in the rate parameter *k*_*A*_, and the carbon usage in the parameter *α*_*C*_. Specifically, with respirofermentative growth represented by the subscript *f* and purely respiratory growth by the subscript *r*, the above considerations require $k_{A_{f}}>k_{A_{r}}$ and $\alpha_{C_{f}}>\alpha_{C_{r}}$.

To obtain a rough estimate of the stoichiometry parameter in respiratory growth $\alpha_{C_{r}}$, we used that proteinogenic amino acids contain approximately four carbon atoms per nitrogen atom. Using molar masses of 12 and 14 g mol^−1^ for carbon and nitrogen, respectively, this ratio translated to (mass-action)

4.20 $$\alpha_{C_{r}}=\frac{4\times 12}{4\times 12+14}=\frac{24}{31}.$$

A further rough estimate, namely that the excess carbon excreted in fermentation is approximately equal in amount to the carbon converted to biomass, gave the stoichiometry in respirofermentative growth as

4.21 $$\alpha_{C_{\;f}}=\frac{8\times 12}{8\times 12+14}=\frac{48}{55}.$$

The full metabolic model, described in §4.1, was explored in figures 3, 4 and 5. The simplified models described earlier were obtained by setting key parameters to zero. For the model with parallel carbon and nitrogen assimilation pathways, the lack of ketoacid recycling or excretion (figure 2) was represented by the ketoacid stoichiometry parameter *γ*_*K*_ = 0. Additionally, the initial ketoacid fraction *k*_0_ was set to zero, and no allocation was made to the ketoacid enzymes, i.e. $f_{K_{\mathrm{ex}}}=f_{K_{\mathrm{re}}}=0$. For the initial core model, which did not distinguish respirofermentative and respiratory growth, $f_{A_{r}}=0$ was additionally forced.

### 4.4. Optimizing ribosomal allocation to maximize steady-state growth rate

We explored the behaviour of the model under parameter modulations that represented different growth environments, while assuming that growth was optimized to suit this environment. The allocation vector f from equation (4.1) was therefore chosen not as a free parameter, but rather as the result of an optimization routine that maximized the growth rate *μ*. The fraction of housekeeping proteins *f*_*Z*_ was excluded from the optimization. The optimization problem can be defined as finding the allocation fraction f that maximizes the growth rate *μ*, while satisfying the allocation constraint

4.22 $$\sum_{i}f_{i}=1.$$

The computation of the optimal allocation fraction is complicated, because the function μ(f) is only defined implicitly: a steady state must be found for each choice of f before the steady-state growth rate can be extracted. The nonlinearity of the Michaelis–Menten kinetics prohibits an explicit solution for *μ* in terms of f, which would be required by gradient-based optimization routines. We were therefore restricted to using a gradient-free optimization routine, for which we chose the Nelder–Mead algorithm as defined in the Optim.jl package [61–63]. We used tolerances of 1.0 × 10^−10^ and set the initial simplex to Optim.AffineSimplexer (*a* = 0.0, *b* = −0.1). The optimization objective was set to minimize the doubling time *t*_*d*_ = log 2/*μ*.

The allocation constraint (4.22) further complicated the optimization procedure. We eliminated one element of the allocation vector by imposing the allocation constraint. However, the Nelder–Mead sampling strategy still allowed for situations where this constrained element became negative. As a practical solution for such situations, and for allocation fractions whose sum exceeded the constraint, we evaluated the doubling time as the inverse of the machine precision, which served as a predefined very large value.

The Nelder–Mead algorithm further proved to be sensitive to numerical inaccuracies when the optimal allocation fraction had zero elements, i.e. the optimal cell entirely lacked expression of some proteins. Therefore, we ran the optimization procedure several times, each with different allocation fractions set to zero and excluded from the optimization. Each iteration resulted in one optimal allocation vector; the one with the largest growth rate (smallest doubling time) was selected as the global optimum.

## Data Availability

The code used to generate the results and figures in this paper has been made available on GitHub (https://www.github.com/istvankleijn/C-GRAM-carbon-nitrogen) and archived on Zenodo [64]. Additional discussion about earlier C-GRAMs, and further details and derivations for the model presented in this paper are provided in electronic supplementary material [65].
